# Quarterly Report (No. 4,) of the Medical and Surgical Practice of the St. George's and St. James's General Dispensary, No. 60, King-Street, Golden-Square; from April 13, to July 12, 1823, Inclusive

**Published:** 1823-08

**Authors:** George Gregory

**Affiliations:** Senior Physician to the Dispeusary, &c.


### STATISTICAL MEDICINE.

ART. I.—*Quarterly Report (No.* 4,*) of the Medical and Surgical Practice of the St. George's and St. James's General Dispensary, No.* 60, *King-street, Golden-square; from April* 13, *to July* 12, 1823, *inclusive.* By GEORGE GREGORY, M.D. Senior Physician to the Dispensary, &c.

| | | | |
|---|---|---|---|
| Acute Constitutional Affections | Fever, Common Continued | 60 | |
| | ——— Remittent, of Children | 19 | |
| | Typhus | 3 | |
| | Variola | 4 | |
| | Varicella | 2 | |
| | Scarlatina | 1 | |
| | Measles | 1 | |
| | Rheumatism, Acute and Subacute | 26 | —116 |
| Diseases of the Head and Nervous System | Phrenitis and Hydrocephalus | 7 | |
| | Epilepsy | 2 | |
| | Mania | 2 | |
| | Palsy | 3 | |
| | Neuralgia | 2 | |
| | Head-ache and Giddiness | 11 | — 27 |
| Diseases of the Thorax | Pneumonia | 11 | |
| | Catarrh and Cynanche | 15 | |
| | Bronchitis, Subacute and Chronic | 24 | |
| | Hæmoptysis and Phthisis | 14 | |
| | Pertussis | 9 | |
| | Diseased Heart | 5 | |
| | Pain of the Side | 8 | — 86 |
| Diseases of the Chylopoietic Viscera | Dyspepsia | 75 | |
| | Colic | 7 | |
| | Diarrhœa | 14 | |
| | Worms | 1 | |
| | Hepatic Affections | 5 | |
| | Piles | 2 | =104 |
| Diseases of the Urinary System | Affection of the Kidney | 1 | |
| | Inflamed Testicle | 1 | |
| | Stricture | 2 | = 4 |
| Diseases of the Uterine System | Irritation from Pregnancy | 8 | |
| | Menorrhagia | 2 | |
| | Abortion | 1 | |
| | Leucorrhœa | 3 | |
| | Hysteria | 3 | |
| | Prolapsus Uteri | 1 | |
| | Diseased Uterus | 2 | |
| | Amenorrhœa | 6 | = 26 |

| | | | |
|---|---|---|---|
| Chronic Constitutional Affections .......... | Scrofula and Diseased Glands .......... | 11 | |
| | Rickets .......... | 1 | |
| | Plethora .......... | 7 | |
| | Cachexia .......... | 2 | |
| | Debility .......... | 10 | |
| | Hypochondriasis .......... | 4 | |
| | Dropsy .......... | 5 | = 40 |
| Chronic Cutaneous Diseases .......... | Lichen .......... | 2 | |
| | Prurigo .......... | 1 | |
| | Porrigo .......... | 9 | |
| | Lepra and Psoriasis .......... | 4 | |
| | Herpes Zoster .......... | 1 | |
| | Pompholyx .......... | 1 | |
| | Urticaria .......... | 3 | |
| | Eruptions not well characterised .......... | 3 | = 24 |
| Syphilitic Affections...... | Primary Symptoms .......... | 4 | |
| | Secondary Symptoms .......... | 8 | |
| | Gonorrhœa .......... | 7 | = 19 |
| Diseases strictly Local .... | Fracture .......... | 1 | |
| | Contusions, Sprains, and slight Injuries .. | 35 | |
| | Phlegmons, Abscesses, and Ulcers ...... | 22 | |
| | Diseases of the Mamma .......... | 5 | |
| | Diseases of Bones .......... | 3 | |
| | Affections of Joints .......... | 10 | |
| | Ophthalmia .......... | 6 | |
| | Herniæ .......... | 2 | |
| | Sore Legs .......... | 10 | |
| | Diseases of the Ear .......... | 2 | |
| | Odontalgia .......... | 1 | = 97 |
| | Cases not reported .......... | | 12 |
| Total .......... | Under the care of the Physicians ........ | 347 | 555 |
| | ———— Surgeons .......... | 150 | |
| | ———— Surgeon-Accoucheur | 58 | |

*Fatal Cases during the Quarter:*—Fever, 1; Variola, 2; Comatose, 1; Phthisis, 2; Pneumonia, 1; Hooping Cough, 1; Jaundice, 1; Incessant Vomiting, 1; Dropsy, 1.—Total, 11.

Febrile affections have taken the lead among the disorders of the last quarter. The greater part of them proved very mild, and yielded in the course of six or eight days to a simple treatment; of which the administration of two or three doses of jalap and calomel constituted the leading, and perhaps only essential part.

The single case of Fever reported as having proved fatal, was that of a man, forty years of age, who, for many years past, had indulged to great excess in spirituous liquors. He died on the thirty-fifth day of fever. On dissection, the lungs were found (particularly in their upper lobes) studded with small vomicæ. The stomach and liver were sound. This adds another to the many proofs which might be adduced in support of the fact, that excess in spirituous liquors predisposes (in this country at least) to *thoracic* much more than to *abdominal* disease.

The fatal case of Jaundice had been under the care of my colleague, Dr. Cloves. On dissection, the pancreas was found greatly enlarged, and pressing on the biliary ducts. This patient died in a state of emaciation, of which it is difficult to convey an adequate idea.

The case of incessant Vomiting noted as proving fatal, was one of those alluded to by Dr. PEMBERTON, in his work on the Diseases of the Abdominal Viscera, p. 132. Examination of the body was not permitted.

The fatal cases of Small-Pox occurred in families where the parents were either obstinately prejudiced against, or indifferent to the advantages of, vaccination. It is gratifying to perceive, however, that there is no indisposition on the part of the great bulk of the people to submit their children to vaccination; and, though the annual deaths in London by small-pox (as reported in the Bills of Mortality,) have not for some years past diminished, they are not sensibly increased. If practitioners will but continue steady in their faith, and discourage all *partial* inoculations for the small-pox, the cause of vaccination cannot fail to prosper.

The case of Pompholyx afforded a striking illustration of all the most interesting features of that rare disease. It occurred in a woman, 73 years of age. The vesicles were of immense size, and scattered over every part of the body, more particularly the legs, thighs, and arms. Many of a smaller size were intermixed with them, of a livid colour; and all of them originated in a degree of cutaneous inflammation, which might be correctly denominated *erratic erysipelas*. The disease produced excessive itching, and upon the legs extensive excoriations, which healed, however, without difficulty. Pompholyx has always been remarked as a disease of peculiar obstinacy; and this case fully corroborated the general opinion. The symptoms first showed themselves on the 20th of April, and did not yield until the first week of July. Purgatives, bark, sarsaparilla, and a variety of other medicines, were tried, but apparently without producing the smallest effect on the disease. The appetite continued unimpaired through its whole course. On one occasion, to relieve a cough which had come on, I took away fourteen ounces of blood from the arm; and this for the time seemed to check the formation of bullæ, but they soon returned. Anasarcous swellings of the legs have since supervened, and symptoms of hydrothorax are now developing themselves. Considering the age of the patient, therefore, no reasonable hopes can be entertained of a favourable issue.

